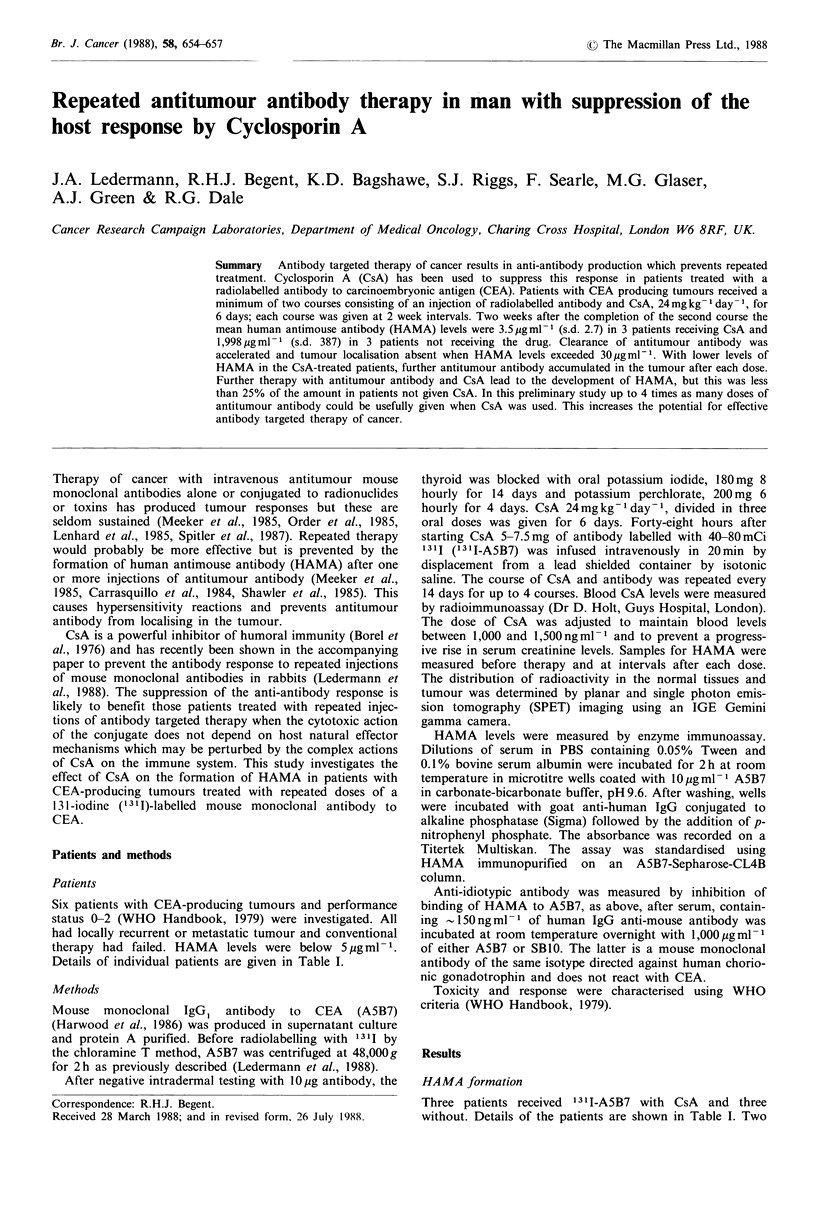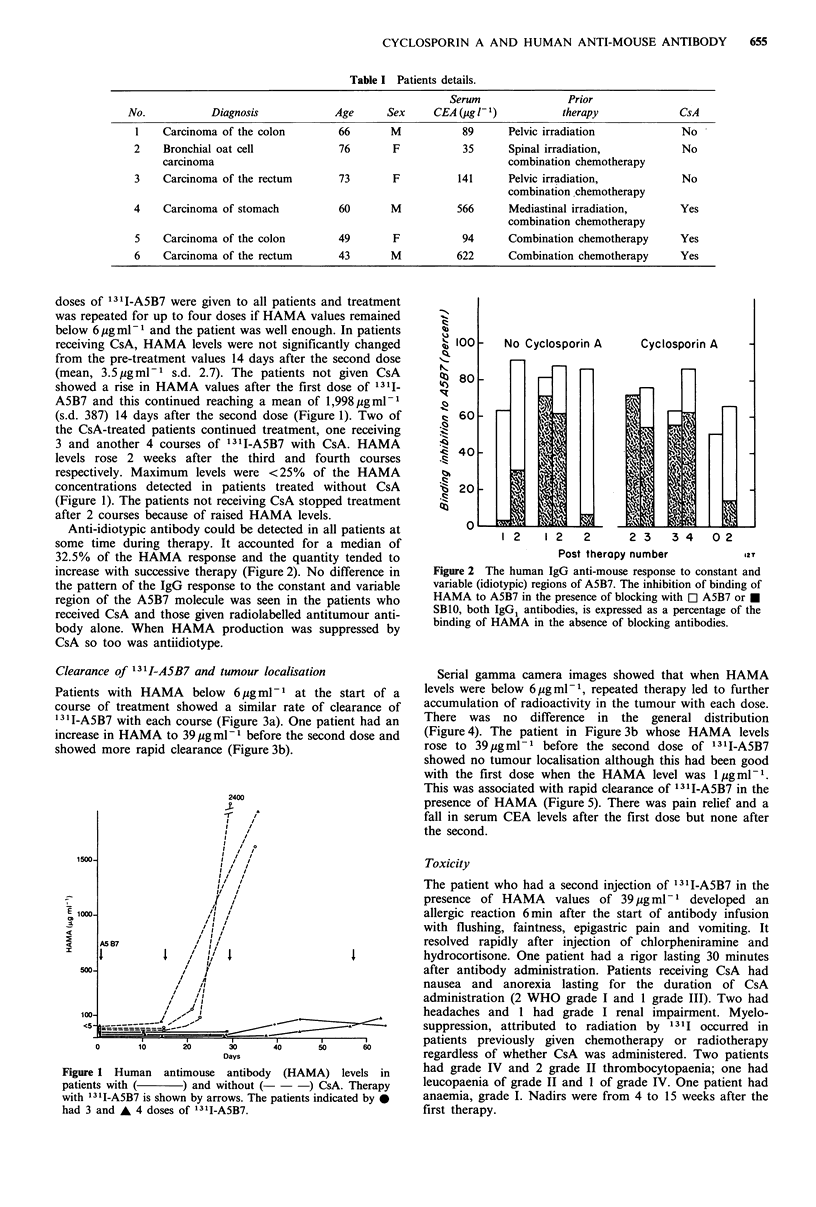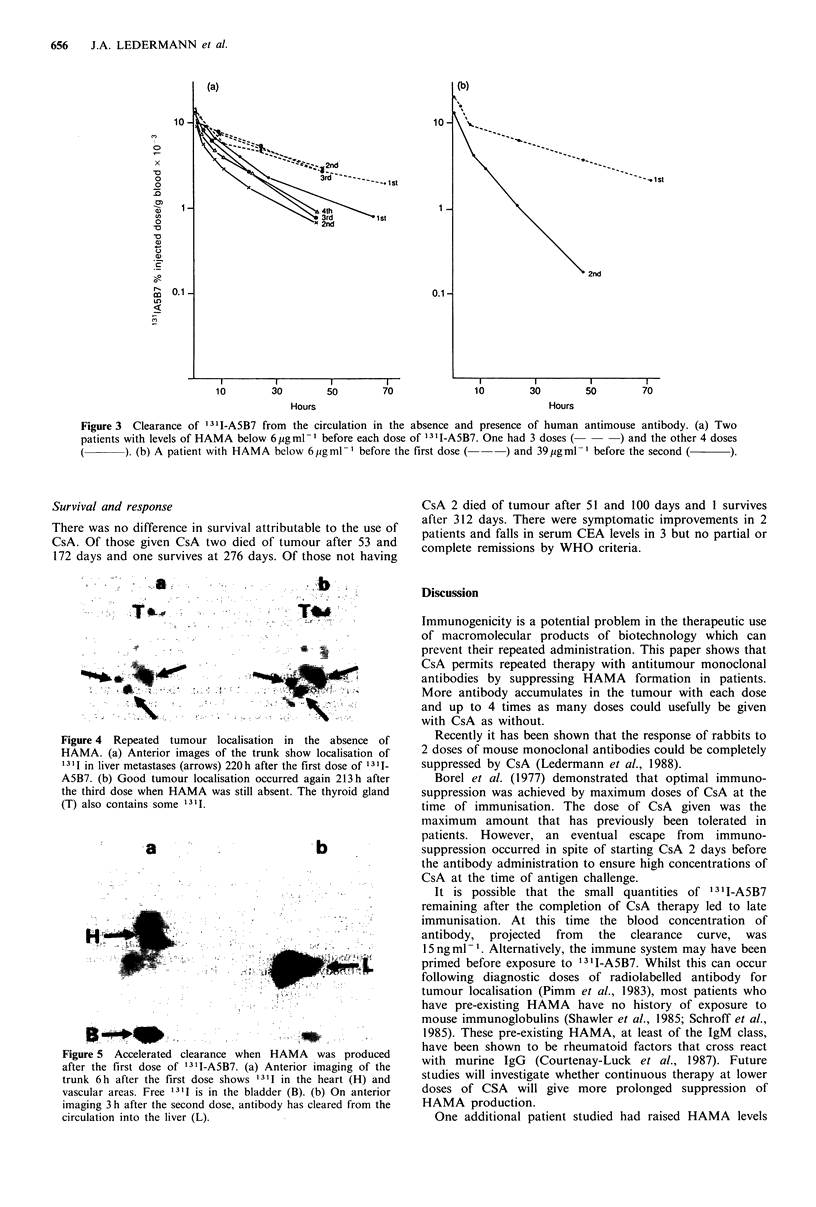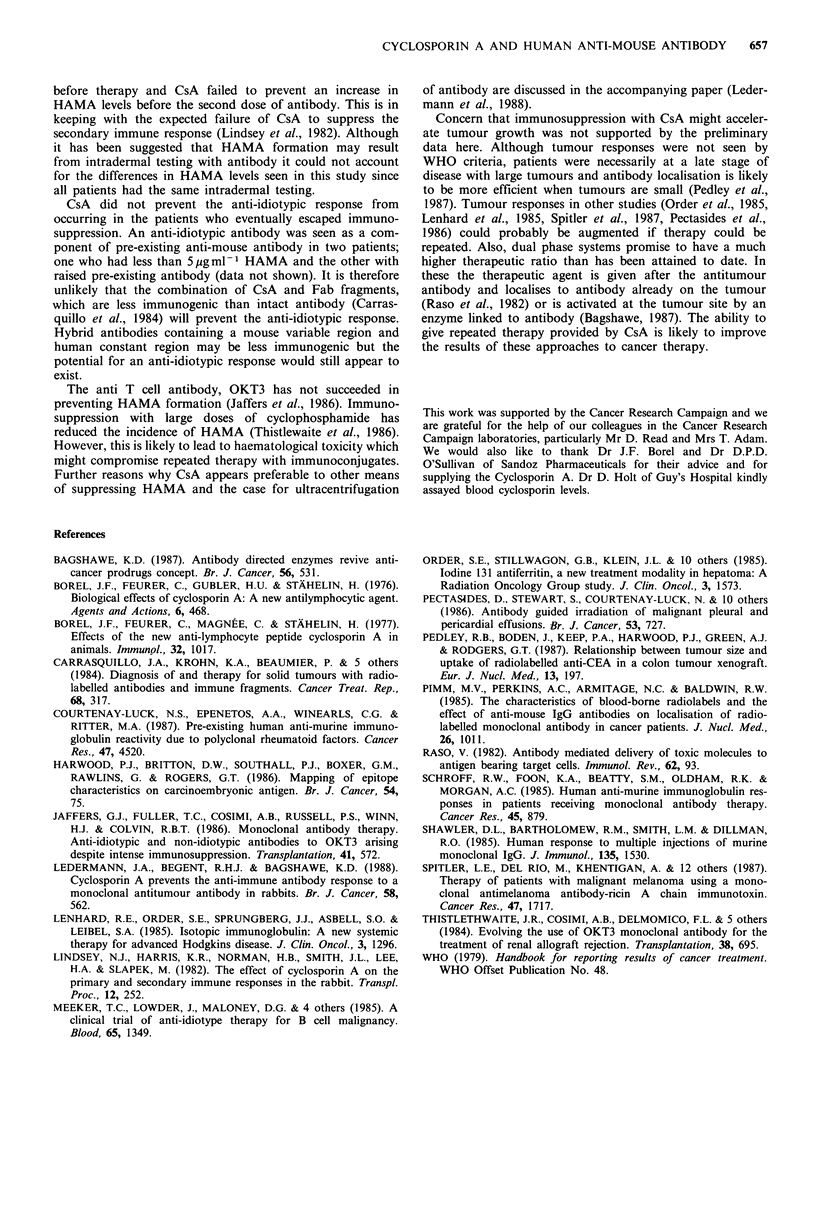# Repeated antitumour antibody therapy in man with suppression of the host response by cyclosporin A.

**DOI:** 10.1038/bjc.1988.279

**Published:** 1988-11

**Authors:** J. A. Ledermann, R. H. Begent, K. D. Bagshawe, S. J. Riggs, F. Searle, M. G. Glaser, A. J. Green, R. G. Dale

**Affiliations:** Department of Medical Oncology, Charing Cross Hospital, London, UK.

## Abstract

**Images:**


*Br. J. Cancer* (1988), **58**, 654–657
© The Macmillan Press Ltd., 1988

### Repeated antitumour antibody therapy in man with suppression of the host response by Cyclosporin A

J.A. Ledermann, R.H.J. Begent, K.D. Bagshawe, S.J. Riggs, F. Searle, M.G. Glaser, A.J. Green & R.G. Dale

*Cancer Research Campaign Laboratories, Department of Medical Oncology, Charing Cross Hospital, London W6 8RF, UK.*

**Summary** Antibody targeted therapy of cancer results in anti-antibody production which prevents repeated treatment. Cyclosporin A (CsA) has been used to suppress this response in patients treated with a radiolabelled antibody to carcinoembryonic antigen (CEA). Patients with CEA producing tumours received a minimum of two courses consisting of an injection of radiolabelled antibody and CsA, $24\,mg\,kg^{-1}\,day^{-1}$, for 6 days; each course was given at 2 week intervals. Two weeks after the completion of the second course the mean human antimouse antibody (HAMA) levels were $3.5\,\mu g\,ml^{-1}$ (s.d. 2.7) in 3 patients receiving CsA and $1{,}998\,\mu g\,ml^{-1}$ (s.d. 387) in 3 patients not receiving the drug. Clearance of antitumour antibody was accelerated and tumour localisation absent when HAMA levels exceeded $30\,\mu g\,ml^{-1}$. With lower levels of HAMA in the CsA-treated patients, further antitumour antibody accumulated in the tumour after each dose. Further therapy with antitumour antibody and CsA lead to the development of HAMA, but this was less than 25% of the amount in patients not given CsA. In this preliminary study up to 4 times as many doses of antitumour antibody could be usefully given when CsA was used. This increases the potential for effective antibody targeted therapy of cancer.

Therapy of cancer with intravenous antitumour mouse monoclonal antibodies alone or conjugated to radionuclides or toxins has produced tumour responses but these are seldom sustained (Meeker *et al.*, 1985, Order *et al.*, 1985, Lenhard *et al.*, 1985, Spitler *et al.*, 1987). Repeated therapy would probably be more effective but is prevented by the formation of human antimouse antibody (HAMA) after one or more injections of antitumour antibody (Meeker *et al.*, 1985, Carrasquillo *et al.*, 1984, Shawler *et al.*, 1985). This causes hypersensitivity reactions and prevents antitumour antibody from localising in the tumour.

CsA is a powerful inhibitor of humoral immunity (Borel *et al.*, 1976) and has recently been shown in the accompanying paper to prevent the antibody response to repeated injections of mouse monoclonal antibodies in rabbits (Ledermann *et al.*, 1988). The suppression of the anti-antibody response is likely to benefit those patients treated with repeated injections of antibody targeted therapy when the cytotoxic action of the conjugate does not depend on host natural effector mechanisms which may be perturbed by the complex actions of CsA on the immune system. This study investigates the effect of CsA on the formation of HAMA in patients with CEA-producing tumours treated with repeated doses of a 131-iodine ($^{131}I$)-labelled mouse monoclonal antibody to CEA.

### Patients and methods

### *Patients*

Six patients with CEA-producing tumours and performance status 0–2 (WHO Handbook, 1979) were investigated. All had locally recurrent or metastatic tumour and conventional therapy had failed. HAMA levels were below $5\,\mu g\,ml^{-1}$. Details of individual patients are given in Table I.

### *Methods*

Mouse monoclonal $IgG_1$ antibody to CEA (A5B7) (Harwood *et al.*, 1986) was produced in supernatant culture and protein A purified. Before radiolabelling with $^{131}I$ by the chloramine T method, A5B7 was centrifuged at 48,000 *g* for 2 h as previously described (Ledermann *et al.*, 1988).

After negative intradermal testing with $10\,\mu g$ antibody, the thyroid was blocked with oral potassium iodide, 180 mg 8 hourly for 14 days and potassium perchlorate, 200 mg 6 hourly for 4 days. CsA $24\,mg\,kg^{-1}\,day^{-1}$, divided in three oral doses was given for 6 days. Forty-eight hours after starting CsA 5–7.5 mg of antibody labelled with 40–80 mCi $^{131}I$ ($^{131}I$-A5B7) was infused intravenously in 20 min by displacement from a lead shielded container by isotonic saline. The course of CsA and antibody was repeated every 14 days for up to 4 courses. Blood CsA levels were measured by radioimmunoassay (Dr D. Holt, Guys Hospital, London). The dose of CsA was adjusted to maintain blood levels between 1,000 and $1{,}500\,ng\,ml^{-1}$ and to prevent a progressive rise in serum creatinine levels. Samples for HAMA were measured before therapy and at intervals after each dose. The distribution of radioactivity in the normal tissues and tumour was determined by planar and single photon emission tomography (SPET) imaging using an IGE Gemini gamma camera.

HAMA levels were measured by enzyme immunoassay. Dilutions of serum in PBS containing 0.05% Tween and 0.1% bovine serum albumin were incubated for 2 h at room temperature in microtitre wells coated with $10\,\mu g\,ml^{-1}$ A5B7 in carbonate-bicarbonate buffer, pH 9.6. After washing, wells were incubated with goat anti-human IgG conjugated to alkaline phosphatase (Sigma) followed by the addition of *p*-nitrophenyl phosphate. The absorbance was recorded on a Titertek Multiskan. The assay was standardised using HAMA immunopurified on an A5B7-Sepharose-CL4B column.

Anti-idiotypic antibody was measured by inhibition of binding of HAMA to A5B7, as above, after serum, containing $\sim 150\,ng\,ml^{-1}$ of human IgG anti-mouse antibody was incubated at room temperature overnight with $1{,}000\,\mu g\,ml^{-1}$ of either A5B7 or SB10. The latter is a mouse monoclonal antibody of the same isotype directed against human chorionic gonadotrophin and does not react with CEA.

Toxicity and response were characterised using WHO criteria (WHO Handbook, 1979).

### Results

### *HAMA formation*

Three patients received $^{131}I$-A5B7 with CsA and three without. Details of the patients are shown in Table I. Two

Correspondence: R.H.J. Begent.
Received 28 March 1988; and in revised form, 26 July 1988.

**Table I** Patients details.

| *No.* | *Diagnosis* | *Age* | *Sex* | *Serum CEA* ($\mu g\, l^{-1}$) | *Prior therapy* | *CsA* |
|---|---|---|---|---|---|---|
| 1 | Carcinoma of the colon | 66 | M | 89 | Pelvic irradiation | No |
| 2 | Bronchial oat cell carcinoma | 76 | F | 35 | Spinal irradiation, combination chemotherapy | No |
| 3 | Carcinoma of the rectum | 73 | F | 141 | Pelvic irradiation, combination chemotherapy | No |
| 4 | Carcinoma of stomach | 60 | M | 566 | Mediastinal irradiation, combination chemotherapy | Yes |
| 5 | Carcinoma of the colon | 49 | F | 94 | Combination chemotherapy | Yes |
| 6 | Carcinoma of the rectum | 43 | M | 622 | Combination chemotherapy | Yes |

doses of $^{131}$I-A5B7 were given to all patients and treatment was repeated for up to four doses if HAMA values remained below 6 $\mu g\, ml^{-1}$ and the patient was well enough. In patients receiving CsA, HAMA levels were not significantly changed from the pre-treatment values 14 days after the second dose (mean, 3.5 $\mu g\, ml^{-1}$ s.d. 2.7). The patients not given CsA showed a rise in HAMA values after the first dose of $^{131}$I-A5B7 and this continued reaching a mean of 1,998 $\mu g\, ml^{-1}$ (s.d. 387) 14 days after the second dose (Figure 1). Two of the CsA-treated patients continued treatment, one receiving 3 and another 4 courses of $^{131}$I-A5B7 with CsA. HAMA levels rose 2 weeks after the third and fourth courses respectively. Maximum levels were <25% of the HAMA concentrations detected in patients treated without CsA (Figure 1). The patients not receiving CsA stopped treatment after 2 courses because of raised HAMA levels.

Anti-idiotypic antibody could be detected in all patients at some time during therapy. It accounted for a median of 32.5% of the HAMA response and the quantity tended to increase with successive therapy (Figure 2). No difference in the pattern of the IgG response to the constant and variable region of the A5B7 molecule was seen in the patients who received CsA and those given radiolabelled antitumour antibody alone. When HAMA production was suppressed by CsA so too was antiidiotype.

**Figure 2** The human IgG anti-mouse response to constant and variable (idiotypic) regions of A5B7. The inhibition of binding of HAMA to A5B7 in the presence of blocking with □ A5B7 or ■ SB10, both $IgG_1$ antibodies, is expressed as a percentage of the binding of HAMA in the absence of blocking antibodies.

### *Clearance of $^{131}$I-A5B7 and tumour localisation*

Patients with HAMA below 6 $\mu g\, ml^{-1}$ at the start of a course of treatment showed a similar rate of clearance of $^{131}$I-A5B7 with each course (Figure 3a). One patient had an increase in HAMA to 39 $\mu g\, ml^{-1}$ before the second dose and showed more rapid clearance (Figure 3b).

**Figure 1** Human antimouse antibody (HAMA) levels in patients with (————) and without (— — —) CsA. Therapy with $^{131}$I-A5B7 is shown by arrows. The patients indicated by ● had 3 and ▲ 4 doses of $^{131}$I-A5B7.

Serial gamma camera images showed that when HAMA levels were below 6 $\mu g\, ml^{-1}$, repeated therapy led to further accumulation of radioactivity in the tumour with each dose. There was no difference in the general distribution (Figure 4). The patient in Figure 3b whose HAMA levels rose to 39 $\mu g\, ml^{-1}$ before the second dose of $^{131}$I-A5B7 showed no tumour localisation although this had been good with the first dose when the HAMA level was 1 $\mu g\, ml^{-1}$. This was associated with rapid clearance of $^{131}$I-A5B7 in the presence of HAMA (Figure 5). There was pain relief and a fall in serum CEA levels after the first dose but none after the second.

### *Toxicity*

The patient who had a second injection of $^{131}$I-A5B7 in the presence of HAMA values of 39 $\mu g\, ml^{-1}$ developed an allergic reaction 6 min after the start of antibody infusion with flushing, faintness, epigastric pain and vomiting. It resolved rapidly after injection of chlorpheniramine and hydrocortisone. One patient had a rigor lasting 30 minutes after antibody administration. Patients receiving CsA had nausea and anorexia lasting for the duration of CsA administration (2 WHO grade I and 1 grade III). Two had headaches and 1 had grade I renal impairment. Myelosuppression, attributed to radiation by $^{131}$I occurred in patients previously given chemotherapy or radiotherapy regardless of whether CsA was administered. Two patients had grade IV and 2 grade II thrombocytopaenia; one had leucopaenia of grade II and 1 of grade IV. One patient had anaemia, grade I. Nadirs were from 4 to 15 weeks after the first therapy.

**Figure 3** Clearance of $^{131}$I-A5B7 from the circulation in the absence and presence of human antimouse antibody. (a) Two patients with levels of HAMA below 6 μg ml$^{-1}$ before each dose of $^{131}$I-A5B7. One had 3 doses (— — —) and the other 4 doses (———). (b) A patient with HAMA below 6 μg ml$^{-1}$ before the first dose (— — —) and 39 μg ml$^{-1}$ before the second (———).

*Survival and response*

There was no difference in survival attributable to the use of CsA. Of those given CsA two died of tumour after 53 and 172 days and one survives at 276 days. Of those not having CsA 2 died of tumour after 51 and 100 days and 1 survives after 312 days. There were symptomatic improvements in 2 patients and falls in serum CEA levels in 3 but no partial or complete remissions by WHO criteria.

**Figure 4** Repeated tumour localisation in the absence of HAMA. (a) Anterior images of the trunk show localisation of $^{131}$I in liver metastases (arrows) 220 h after the first dose of $^{131}$I-A5B7. (b) Good tumour localisation occurred again 213 h after the third dose when HAMA was still absent. The thyroid gland (T) also contains some $^{131}$I.

**Figure 5** Accelerated clearance when HAMA was produced after the first dose of $^{131}$I-A5B7. (a) Anterior imaging of the trunk 6 h after the first dose shows $^{131}$I in the heart (H) and vascular areas. Free $^{131}$I is in the bladder (B). (b) On anterior imaging 3 h after the second dose, antibody has cleared from the circulation into the liver (L).

### Discussion

Immunogenicity is a potential problem in the therapeutic use of macromolecular products of biotechnology which can prevent their repeated administration. This paper shows that CsA permits repeated therapy with antitumour monoclonal antibodies by suppressing HAMA formation in patients. More antibody accumulates in the tumour with each dose and up to 4 times as many doses could usefully be given with CsA as without.

Recently it has been shown that the response of rabbits to 2 doses of mouse monoclonal antibodies could be completely suppressed by CsA (Ledermann et al., 1988).

Borel et al. (1977) demonstrated that optimal immunosuppression was achieved by maximum doses of CsA at the time of immunisation. The dose of CsA given was the maximum amount that has previously been tolerated in patients. However, an eventual escape from immunosuppression occurred in spite of starting CsA 2 days before the antibody administration to ensure high concentrations of CsA at the time of antigen challenge.

It is possible that the small quantities of $^{131}$I-A5B7 remaining after the completion of CsA therapy led to late immunisation. At this time the blood concentration of antibody, projected from the clearance curve, was 15 ng ml$^{-1}$. Alternatively, the immune system may have been primed before exposure to $^{131}$I-A5B7. Whilst this can occur following diagnostic doses of radiolabelled antibody for tumour localisation (Pimm et al., 1983), most patients who have pre-existing HAMA have no history of exposure to mouse immunoglobulins (Shawler et al., 1985; Schroff et al., 1985). These pre-existing HAMA, at least of the IgM class, have been shown to be rheumatoid factors that cross react with murine IgG (Courtenay-Luck et al., 1987). Future studies will investigate whether continuous therapy at lower doses of CSA will give more prolonged suppression of HAMA production.

One additional patient studied had raised HAMA levels before therapy and CsA failed to prevent an increase in HAMA levels before the second dose of antibody. This is in keeping with the expected failure of CsA to suppress the secondary immune response (Lindsey *et al.*, 1982). Although it has been suggested that HAMA formation may result from intradermal testing with antibody it could not account for the differences in HAMA levels seen in this study since all patients had the same intradermal testing.

CsA did not prevent the anti-idiotypic response from occurring in the patients who eventually escaped immunosuppression. An anti-idiotypic antibody was seen as a component of pre-existing anti-mouse antibody in two patients; one who had less than $5 \mu g\, ml^{-1}$ HAMA and the other with raised pre-existing antibody (data not shown). It is therefore unlikely that the combination of CsA and Fab fragments, which are less immunogenic than intact antibody (Carrasquillo *et al.*, 1984) will prevent the anti-idiotypic response. Hybrid antibodies containing a mouse variable region and human constant region may be less immunogenic but the potential for an anti-idiotypic response would still appear to exist.

The anti T cell antibody, OKT3 has not succeeded in preventing HAMA formation (Jaffers *et al.*, 1986). Immunosuppression with large doses of cyclophosphamide has reduced the incidence of HAMA (Thistlewaite *et al.*, 1986). However, this is likely to lead to haematological toxicity which might compromise repeated therapy with immunoconjugates. Further reasons why CsA appears preferable to other means of suppressing HAMA and the case for ultracentrifugation of antibody are discussed in the accompanying paper (Ledermann *et al.*, 1988).

Concern that immunosuppression with CsA might accelerate tumour growth was not supported by the preliminary data here. Although tumour responses were not seen by WHO criteria, patients were necessarily at a late stage of disease with large tumours and antibody localisation is likely to be more efficient when tumours are small (Pedley *et al.*, 1987). Tumour responses in other studies (Order *et al.*, 1985, Lenhard *et al.*, 1985, Spitler *et al.*, 1987, Pectasides *et al.*, 1986) could probably be augmented if therapy could be repeated. Also, dual phase systems promise to have a much higher therapeutic ratio than has been attained to date. In these the therapeutic agent is given after the antitumour antibody and localises to antibody already on the tumour (Raso *et al.*, 1982) or is activated at the tumour site by an enzyme linked to antibody (Bagshawe, 1987). The ability to give repeated therapy provided by CsA is likely to improve the results of these approaches to cancer therapy.

This work was supported by the Cancer Research Campaign and we are grateful for the help of our colleagues in the Cancer Research Campaign laboratories, particularly Mr D. Read and Mrs T. Adam. We would also like to thank Dr J.F. Borel and Dr D.P.D. O'Sullivan of Sandoz Pharmaceuticals for their advice and for supplying the Cyclosporin A. Dr D. Holt of Guy's Hospital kindly assayed blood cyclosporin levels.

### References

BAGSHAWE, K.D. (1987). Antibody directed enzymes revive anti-cancer prodrugs concept. *Br. J. Cancer*, **56**, 531.

BOREL, J.F., FEURER, C., GUBLER, H.U. & STÄHELIN, H. (1976). Biological effects of cyclosporin A: A new antilymphocytic agent. *Agents and Actions*, **6**, 468.

BOREL, J.F., FEURER, C., MAGNÉE, C. & STÄHELIN, H. (1977). Effects of the new anti-lymphocyte peptide cyclosporin A in animals. *Immunol.*, **32**, 1017.

CARRASQUILLO, J.A., KROHN, K.A., BEAUMIER, P. & 5 others (1984). Diagnosis of and therapy for solid tumours with radio-labelled antibodies and immune fragments. *Cancer Treat. Rep.*, **68**, 317.

COURTENAY-LUCK, N.S., EPENETOS, A.A., WINEARLS, C.G. & RITTER, M.A. (1987). Pre-existing human anti-murine immunoglobulin reactivity due to polyclonal rheumatoid factors. *Cancer Res.*, **47**, 4520.

HARWOOD, P.J., BRITTON, D.W., SOUTHALL, P.J., BOXER, G.M., RAWLINS, G. & ROGERS, G.T. (1986). Mapping of epitope characteristics on carcinoembryonic antigen. *Br. J. Cancer*, **54**, 75.

JAFFERS, G.J., FULLER, T.C., COSIMI, A.B., RUSSELL, P.S., WINN, H.J. & COLVIN, R.B.T. (1986). Monoclonal antibody therapy. Anti-idiotypic and non-idiotypic antibodies to OKT3 arising despite intense immunosuppression. *Transplantation*, **41**, 572.

LEDERMANN, J.A., BEGENT, R.H.J. & BAGSHAWE, K.D. (1988). Cyclosporin A prevents the anti-immune antibody response to a monoclonal antitumour antibody in rabbits. *Br. J. Cancer*, **58**, 562.

LENHARD, R.E., ORDER, S.E., SPRUNGBERG, J.J., ASBELL, S.O. & LEIBEL, S.A. (1985). Isotopic immunoglobulin: A new systemic therapy for advanced Hodgkins disease. *J. Clin. Oncol.*, **3**, 1296.

LINDSEY, N.J., HARRIS, K.R., NORMAN, H.B., SMITH, J.L., LEE, H.A. & SLAPEK, M. (1982). The effect of cyclosporin A on the primary and secondary immune responses in the rabbit. *Transpl. Proc.*, **12**, 252.

MEEKER, T.C., LOWDER, J., MALONEY, D.G. & 4 others (1985). A clinical trial of anti-idiotype therapy for B cell malignancy. *Blood*, **65**, 1349.

ORDER, S.E., STILLWAGON, G.B., KLEIN, J.L. & 10 others (1985). Iodine 131 antiferritin, a new treatment modality in hepatoma: A Radiation Oncology Group study. *J. Clin. Oncol.*, **3**, 1573.

PECTASIDES, D., STEWART, S., COURTENAY-LUCK, N. & 10 others (1986). Antibody guided irradiation of malignant pleural and pericardial effusions. *Br. J. Cancer*, **53**, 727.

PEDLEY, R.B., BODEN, J., KEEP, P.A., HARWOOD, P.J., GREEN, A.J. & RODGERS, G.T. (1987). Relationship between tumour size and uptake of radiolabelled anti-CEA in a colon tumour xenograft. *Eur. J. Nucl. Med.*, **13**, 197.

PIMM, M.V., PERKINS, A.C., ARMITAGE, N.C. & BALDWIN, R.W. (1985). The characteristics of blood-borne radiolabels and the effect of anti-mouse IgG antibodies on localisation of radio-labelled monoclonal antibody in cancer patients. *J. Nucl. Med.*, **26**, 1011.

RASO, V. (1982). Antibody mediated delivery of toxic molecules to antigen bearing target cells. *Immunol. Rev.*, **62**, 93.

SCHROFF, R.W., FOON, K.A., BEATTY, S.M., OLDHAM, R.K. & MORGAN, A.C. (1985). Human anti-murine immunoglobulin responses in patients receiving monoclonal antibody therapy. *Cancer Res.*, **45**, 879.

SHAWLER, D.L., BARTHOLOMEW, R.M., SMITH, L.M. & DILLMAN, R.O. (1985). Human response to multiple injections of murine monoclonal IgG. *J. Immunol.*, **135**, 1530.

SPITLER, L.E., DEL RIO, M., KHENTIGAN, A. & 12 others (1987). Therapy of patients with malignant melanoma using a monoclonal antimelanoma antibody-ricin A chain immunotoxin. *Cancer Res.*, **47**, 1717.

THISTLETHWAITE, J.R., COSIMI, A.B., DELMOMICO, F.L. & 5 others (1984). Evolving the use of OKT3 monoclonal antibody for the treatment of renal allograft rejection. *Transplantation*, **38**, 695.

WHO (1979). *Handbook for reporting results of cancer treatment.* WHO Offset Publication No. 48.